# Phytochemicals for the Prevention and Treatment of Gastric Cancer: Effects and Mechanisms

**DOI:** 10.3390/ijms21020570

**Published:** 2020-01-16

**Authors:** Qian-Qian Mao, Xiao-Yu Xu, Ao Shang, Ren-You Gan, Ding-Tao Wu, Atanas G. Atanasov, Hua-Bin Li

**Affiliations:** 1 Guangdong Provincial Key Laboratory of Food, Nutrition and Health, Department of Nutrition, School of Public Health, Sun Yat-sen University, Guangzhou 510080, China; maoqq@mail2.sysu.edu.cn (Q.-Q.M.); xuxy53@mail2.sysu.edu.cn (X.-Y.X.); shangao@mail2.sysu.edu.cn (A.S.); 2 Research Center for Plants and Human Health, Institute of Urban Agriculture, Chinese Academy of Agricultural Sciences, Chengdu 610213, China; 3 Department of Food Science & Technology, School of Agriculture and Biology, Shanghai Jiao Tong University, Shanghai 200240, China; 4 Institute of Food Processing and Safety, College of Food Science, Sichuan Agricultural University, Ya’an 625014, China; DT_Wu@sicau.edu.cn; 5 Department of Molecular Biology, Institute of Genetics and Animal Breeding of the Polish Academy of Sciences, Jastrzebiec, 05-552 Magdalenka, Poland; atanas.atanasov@univie.ac.at; 6 Institute of Neurobiology, Bulgarian Academy of Sciences, 23 Acad. G. Bonchev str., 1113 Sofia, Bulgaria; 7 Department of Pharmacognosy, University of Vienna, 1090 Vienna, Austria; 8 Ludwig Boltzmann Institute for Digital Health and Patient Safety, Medical University of Vienna, Spitalgasse 23, 1090 Vienna, Austria

**Keywords:** phytochemicals, gastric cancer, anticancer, mechanism of action

## Abstract

Gastric cancer is the fifth most common cancer, and the third most prevalent cause of cancer-related deaths in the world. Voluminous evidence has demonstrated that phytochemicals play a critical role in the prevention and management of gastric cancer. Most epidemiological investigations indicate that the increased intake of phytochemicals could reduce the risk of gastric cancer. Experimental studies have elucidated the mechanisms of action, including inhibiting cancer cell proliferation, inducing apoptosis and autophagy, and suppressing angiogenesis as well as cancer cell metastasis. These mechanisms have also been related to the inhibition of *Helicobacter pylori* and the modulation of gut microbiota. In addition, the intake of phytochemicals could enhance the efficacy of anticancer chemotherapeutics. Moreover, clinical studies have illustrated that phytochemicals have the potential for the prevention and the management of gastric cancer in humans. To provide an updated understanding of relationships between phytochemicals and gastric cancer, this review summarizes the effects of phytochemicals on gastric cancer, highlighting the underlying mechanisms. This review could be helpful for guiding the public in preventing gastric cancer through phytochemicals, as well as in developing functional food and drugs for the prevention and treatment of gastric cancer.

## 1. Introduction

According to the data from the World Health Organization in 2015, cancer has become an important cause of premature death in many countries [1]. Gastric cancer is the fifth most commonly diagnosed cancer in the world, and its mortality ranks third in cancers, with an estimated 783,000 deaths in 2018 [1,2]. Due to the high incidence and mortality rate, gastric cancer is considered a severe public health problem [3]. According to the anatomy of stomach, gastric cancer can be classified into cardia and noncardia gastric cancer. In histopathology, gastric cancer can be categorized into intestinal-type and diffuse-type [4]. *Helicobacter pylori* infection, high salt intake and smoking are considered to be the main risk factors for gastric cancer worldwide. In Europe, the amplification of *HER-2* gene was found to be a risk factor [5]. In Asia, a study revealed that ethnicity plays a role in the onset of gastric cancer, and Chinese race was more susceptible to the cancer [6]. To date, chemotherapy, radiation therapy, and gastrectomy have been recognized as the main therapies for treating gastric cancer [7]. However, these therapies usually cause severe side effects or toxicity, thus restricting their application [8,9]. Additionally, the resistance of anticancer drugs also limits the success rate of chemotherapy [10]. Thus, it is urgent and necessary to find a more effective and less toxic strategy for the prevention and management of gastric cancer.

**Figure 1:**
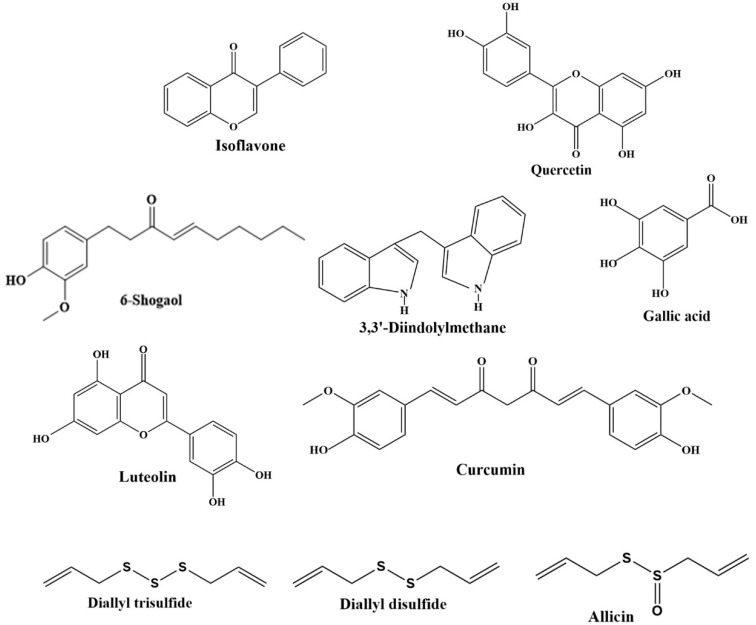
Chemical structures of several phytochemicals against gastric cancer.

Diet plays a prominent role in gastric cancer prevention and management [11]. Increasing evidence from epidemiological studies indicated that natural dietary products have anticancer activity, such as fruits, vegetables, spices, soy, cereals, and edible macro-fungi [12,13,14,15]. Furthermore, many studies found that the risk of gastric cancer was inversely associated with the intake of natural products [16]. The beneficial effects of these natural products could be attributed to the phytochemicals [17,18,19], and the chemical structures of several phytochemicals are showed in Figure 1. In addition, experimental studies indicated that phytochemicals exhibited protective effects against gastric cancer through several mechanisms, including inhibition of cell proliferation [20], induction of apoptosis [21] and autophagy [22], anti-angiogenesis [23], suppression of cell metastasis [24], modulation of gut microbiota [25], and inhibition of *Helicobacter pylori* [26]. Moreover, the use of phytochemicals could be a promising adjuvant therapy for gastric cancer. This review aims to summarize the effects of phytochemicals on the prevention and management of gastric cancer, with the mechanisms of action intensively discussed, and it also illustrates the bioavailability and safety of phytochemicals.

## 2. Epidemiological Studies

Numerous epidemiological studies have demonstrated that the consumption of natural dietary products is essential to the prevention and management of gastric cancer [27,28]. A case-control study reported that the consumption of fresh fruits and vegetables could reduce the risk of gastric cancer with an odds ratio (OR) of 0.15 (95% CI, 0.04–0.64) [6]. In addition, the frequent intake of citrus fruits, vegetables, legumes, garlic, and olive oil showed protective effects against gastric cancer [29]. Additionally, the consumption of garlic, onion, and citrus fruits was reported to decrease the risk of gastric cancer with ORs of 0.35 (95% CI, 0.13–0.95), 0.34 (95% CI, 0.19–0.62), and 0.31 (95% CI, 0.17–0.59), respectively [30]. A meta-analysis also found that the high intake of citrus fruits could reduce the risk of gastric cancer (OR, 0.72; 95% CI, 0.64–0.81) [31]. Moreover, the increased intake of mushroom and soybean products was associated with a lower risk of gastric cancer with OR of 0.30 (95% CI, 0.15–0.62) and 0.35 (95% CI, 0.16–0.75), respectively [32].

Several cohort studies also reported that the intake of fresh fruits and vegetables was inversely associated with the risk of gastric cancer [33,34]. The intake of total plant food, including whole grains, vegetables, and citrus fruit, was negatively related to gastric cancer risk in men (RR, 0.79; 95% Cl, 0.67–0.93) [35]. Furthermore, higher consumption of brassica vegetables and citrus fruits was correlated with a decreased risk of gastric noncardia cancer with RRs of 0.51 (95% CI, 0.28–0.92) and 0.38 (95% CI, 0.21–0.69), respectively [34]. In addition, a meta-analysis revealed that high intake of allium vegetables could decrease the risk of gastric cancer (OR, 0.54; 95% CI, 0.43–0.65) [36]. Additionally, a decrease in gastric cancer risk was observed with increased intake of yellow vegetable and white vegetable with ORs of 0.64 (95% Cl, 0.45–0.92) and 0.48 (95% Cl, 0.25–0.89), respectively [33]. High consumption of green and yellow vegetables was associated with lower mortality of gastric cancer (RR, 0.4; 95% CI, 0.2–0.9) [34]. Moreover, soy products also had a protective effect against gastric cancer [18,37]. A prospective study suggested that the intake of total soy products could decrease the risk of gastric cancer death with hazard ratio (HR) of 0.5 (95% CI, 0.26–0.93) [28]. Additionally, the consumption of tofu was inversely associated with distal gastric cancer risk in men (HR, 0.64; 95% CI, 0.42–0.99), and the high intake of dry bean showed a protective effect against gastric cancer in postmenopausal women (HR, 0.63; 95% CI, 0.43–0.91) [38].

The phytochemicals in the dietary natural products played a critical role in reducing the risk of gastric cancer. For example, a case-control study suggested that the intake of total quercetin in foods and beverages was reversely related to the risk of noncardiac gastric adenocarcinoma, with an adjusted OR of 0.57 (95% CI, 0.40–0.83) [19]. Additionally, a nested case-control study revealed that the increased plasma level of β-carotene mainly from fruits and vegetables was associated with the reduced risk of gastric cancer (OR, 0.46; 95% CI, 0.28–0.75) [39]. Another study showed that the concentration of isoflavones in serum was negatively related to gastric cancer risk [37]. Moreover, the increased intake of total dietary flavonoids and lycopene was related to the decreased risk of gastric cancer with ORs of 0.49 (95% CI, 0.31–0.76) and 0.60 (95% CI, 0.42–0.85), respectively [40,41]. Furthermore, a high intake of anthocyanidins presented a reduction in the mortality of gastric cardia cancer (HR, 0.63; 95% CI, 0.42–0.95) [42]. In addition, another study found that isothiocyanates were effective in protecting against gastric cancer, particularly among those who were lack of genes *GSTMI* (glutathione S-transferase M1) and *GSTTI* (glutathione S-transferase T1) (OR, 0.44; 95% CI, 0.21–0.93) [43].

However, there are inconsistent results in some epidemiological studies regarding the association between the consumption of fruits and vegetables and gastric cancer risk [44,45]. A cohort study demonstrated that the intake of fruits was not significantly correlated with the risk of gastric cancer, while high consumption of green leafy vegetables and root vegetables significantly reduced the risk of gastric cancer with HRs of 0.64 (95% CI, 0.42–0.99) and 0.43 (95% CI, 0.27–0.69), respectively [46]. Additionally, a pooled analysis of four cohort studies demonstrated that the total vegetable consumption was inversely related to distal gastric cancer risk in men (multivariate HR, 0.78; 95%CI, 0.63–0.97), whereas there was no association between total fruit intake and the risk of gastric cancer [44]. Furthermore, an inverse association was observed between fruit consumption and distal gastric cancer risk in men (HR, 0.50; 95% CI, 0.29–0.84), while no relation was found in women [47]. In a prospective study, citrus fruit intake could decrease the risk of gastric cardia cancer, but the intake of vegetables was not related to the risk of gastric cancer [45]. Furthermore, evidence from cohort studies pointed out that the consumption of garlic was not correlated with gastric cancer risk, with a pooled multivariable RR of 1.39 (95% CI, 0.89–2.17) [48]. Additionally, it was inconsistent with the effects of some phytochemicals on the incidence of gastric cancer. The intake of isoflavone or flavonoid showed no relationship with gastric cancer risk [49,50]. Moreover, no association was found between the intake of carotenoids and the risk of gastric cancer [51]. The inconsistent results might be due to the consumed levels of phytochemicals and the differences in regions, dietary, and lifestyles, as well as the data accessing methods [37].

Overall, most epidemiological investigations have suggested that the consumption of natural dietary products is inversely associated with the risk of gastric cancer (Table 1). The protective effects of natural dietary products against gastric cancer could be attributed to the phytochemicals. However, several cohort studies have found that the intake of some vegetables, fruits, and phytochemicals had no effects on gastric cancer. Thus, more epidemiological studies with better design and quality control are needed in the future.

## 3. Experimental Studies

The effects of phytochemicals against gastric cancer have been extensively investigated, and the mechanisms of action have been also explored. These anti-cancer effects and mechanisms will be intensively discussed below.

### 3.1. Inhibition of Cell Proliferation

It has been well documented that various phytochemicals can inhibit the proliferation of human gastric cancer cells and the growth of gastric tumors in mice. In several in vitro studies, allitridi [52], mycelia and polysaccharides of a mushroom [20], labdane diterpenes in *Curcuma mangga* rhizomes [53], poncirin [54], and apigenin [55] were found to inhibit the proliferation of human gastric cancer cell lines. Additionally, the extract of ramson could arrest AGS human gastric cancer cells in G_2_/M phase via the downregulation of cyclin B, resulting in the inhibition of proliferation [56]. Additionally, diallyl disulfide isolated from garlic could arrest MGC803 human gastric cancer cells at the G_2_/M phase by activating the expression of checkpoint kinase-1(Chk1), as well as ataxia telangiectasia and Rad3-related (ATR) protein kinases, and decreasing the expression of cell division cycle 25C (CDC25C) and cyclin B1 [57]. Another study found that the activation of p38 mitogen-activated protein kinase (MAPK) pathway was involved in diallyl disulfide-induced G_2_/M arrest [58]. It could also induce the differentiation of MGC803 cells by decreasing the phosphorylation of extracellular signal-regulated kinase (ERK1/2) protein [59]. Furthermore, diallyl trisulfide, a garlic organosulfide showed an antiproliferative effect on AGS cells by inducing mitotic arrest with increased expression of cyclin B1 and tumor suppressor p53 [60]. Moreover, latcripin 1 from a mushroom had an antiproliferative effect against SGC-7901 and BGC-823 gastric cancer cells by arresting cells at the S phase [61]. Furthermore, myricetin exhibited an antiproliferative effect against HGC-27 and SGC7901 cells by downregulating the expression of cyclinB1, cyclinD1, CDK1, and CDC25C [62]. An in vivo study pointed out that S-allylmercaptocysteine, one of the garlic derivatives, could suppress the growth of SGC-7901 xenografts in BALB/c nude mice [63]. In addition, 6-shogaol from ginger inhibited the gastric tumor growth in athymic nude mice, and it was also found to inhibit the viability of gastric cancer cells, damage microtubules and induce mitotic arrest [64]. Furthermore, (-)-epigallocatechin gallate (EGCG) inhibited the proliferation of SGC-7901 gastric cancer cells and the growth of gastric tumors in mice by suppressing Wnt/β-catenin signaling [65].

### 3.2. Induction of Apoptosis

**Figure 2:**
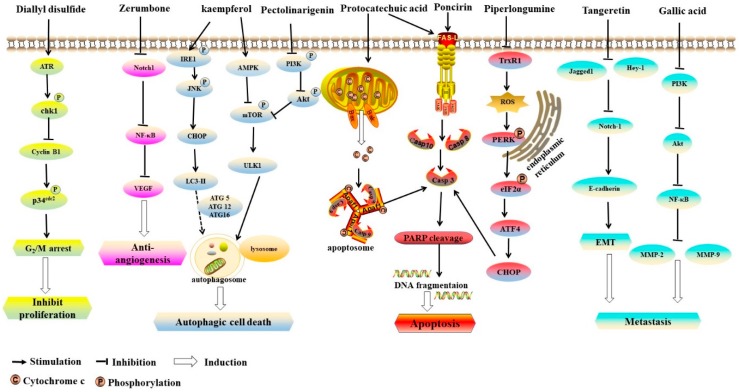
The anticancer mechanisms of phytochemicals on gastric cancer. Diallyl disulfide induced G_2_/M arrest by activating MAPK pathway. Zerumbone showed anti-angiogenesis activity via the inhibition of Notch1/NF-κB/VEGF pathway. Kaempferol induced autophagic cell death via IRE1/JNK/CHOP and AMPK/ULK1 pathways. Pectolinarigenin induced autophagic cell death via PI3K/Akt/mTOR pathway. Poncirin induced apoptosis via the death receptor pathway. Piperlongumine induced apoptosis via ROS-triggered ER-stress and mitochondrial dysfunction, while protocatechuic acid induced apoptosis either through Fas/FasL death receptor or mitochondrial pathways. Tangeretin inhibited migration and invasion by reducing the expressions of Notch-1, Jagged1/2 and Hey-1. Gallic acid could suppress metastasis by downregulating PI3K/Akt pathway.

Induction of apoptosis has been found to be a pivotal mechanism of the inhibition on the initiation and the development of cancer [66,67,68]. It was found that protocatechuic acid could induce the apoptosis of AGS cells through Fas/Fas ligand (FasL) death receptor or mitochondrial pathways accompanied with phosphorylation of c-Jun N-terminal kinase (JNK), p38 mitogen-activating protein kinases (MAPK), and p53 [69]. Additionally, poncirin, rich in citrus fruits, could induce apoptosis in AGS cells via death receptor pathway with increased level of FasL protein, activation of Caspase-8 and Caspase-3, and cleavage of poly (ADP-ribose) polymerase (PARP) [70]. Additionally, the treatment of *Citrus reticulata* Blanco extract could increase apoptosis in SNU-668 human gastric cancer cells through upregulating the expression of B-cell lymphoma 2 (Bcl-2)-associated X protein (Bax) and Caspase-3 [71]. Furthermore, an in vitro study demonstrated that α-mangostin isolated from the pericarp of mangosteen induced apoptosis of BGC-823 and SGC-7901 human gastric cancer cell lines via the reduction of the mitochondrial membrane potential, and the suppression of STAT3 signaling pathway with decreased B-cell lymphoma-extralarge (Bcl-xL) and apoptosis regulator Mcl-1 protein levels [72]. In human gastric signet ring carcinoma cells, the extract of dried ripe fruit of *Vitex agnus-castus* induced apoptosis via intracellular oxidative stress and mitochondrial membrane damage [73]. Moreover, hispolon, a phenolic compound of *Phellinus linteus*, exhibited cytotoxic activity against human gastric cancer cells but not normal gastric cells via the induction of apoptosis, associated with the mitochondrial pathway [74]. Furthermore, the black soybean extracts induced apoptosis of AGS cells in a dose-dependent manner by increasing the levels of Bax and Caspase-3, as well as the cleavage of PARP [75]. It was found that piperlongumine (isolated from the fruit of long pepper) inhibited the activity of thioredoxin reductase 1 (TrxR1), resulting in the induction of apoptosis in human gastric cancer cells via reactive oxygen species (ROS)-triggered ER-stress and mitochondrial dysfunction [21]. In addition, it was observed that allitridi could lead to apoptosis by decreasing the expression of Bcl-2 and increasing the level and activity of Caspase-3 in BGC823 human gastric cancer cell line [52]. Furthermore, it was found that catechin extract and EGCG of green tea [76], theaflavins of black tea [77], and polyphenol extract of oolong tea [78] could induce apoptosis in KATO III human gastric cancer cells. In murine gastric cancer syngeneic model, bamboo-shaving polysaccharides inhibited tumor growth and prolonged the survival of mice bearing a gastric tumor by inducing tumor cell apoptosis [79]. Accumulating evidence has suggested that phytochemicals can induce apoptosis of gastric cancer cells mainly through death receptors or mitochondrial pathways (Figure 2).

### 3.3. Autophagy

Autophagy is an important process of intracellular material renewal and recycle. Some damaged proteins or organelles are engulfed by autophagosomes and sent to autolysosomes for degradation [80]. It has been demonstrated that autophagy plays a dual role in the development of cancer [22,81]. On one hand, under most conditions, autophagy can induce autophagic cancer cell death. On the other hand, autophagy can suppress apoptosis, contributing to the survival of cancer cells sometimes. In gastric cancer cells, the treatment of kaempferol, a natural flavonoid, induced autophagic cell death via inositol-requiring-1 (IRE1)/JNK/-CCAAT-enhancer-binding protein homologous protein (CHOP), AMPK/UNC-51-like autophagy activating kinase 1 (ULK1), and histone deacetylase (HDAC)/G9a (a histone lysine 9 dimethylation-specific methyltransferase) pathways [22]. Additionally, pectolinarigenin extracted from *Cirsium chanroenicum* showed anticancer activity by inducing autophagy in AGS and MKN-28 human gastric cancer cells, mainly through the downregulation of phoshatidylinositol-3-kinase (PI3K)/protein kinase B (Akt)/mammalian target of rapamycin (mTOR) pathway [82]. In addition, 3,3’-diindolylmethane isolated from cruciferous vegetables increased the expression of autophagy-related 5 (ATG5) and microtubule associated protein light chain 3 (LC3) in gastric cancer cells and decreased the level of microRNA-30e, which targets gene *ATG5* to inhibit its translation [83]. Moreover, the treatment of latcripin 1 protein present in *Lentinula edodes* could lead to autophagy in SGC-7901 and BGC-823 gastric cancer cell lines accompanied with the formation of autophagosomes via the change of LC3I into LC3II [61]. Furthermore, perillaldehyde isolated from *Perilla frutescens* displayed anticancer effects against gastric cancer both in vitro and in vivo. In MFCs mouse and GC9811-P human gastric cancer cells, perillaldehyde increased the phosphorylation of AMPK, leading to autophagy in the cells [84]. In mice bearing gastric tumor, perilaldehyde treatment inhibited the growth of the gastric tumor and upregulated the levels of autophagy-associated proteins, such as beclin-1, LC3-II, and cathepsin. However, it was found that quercetin induced protective autophagy against the apoptosis of AGS and MKN-28 gastric cancer cells, suggesting that autophagy could contribute to the survival of cancer cells in certain circumstances [85].

### 3.4. Inhibition of Tumor Angiogenesis

It has been reported that angiogenesis is critical for tumor growth and survival prognosis of gastric cancer [86]. Vascular endothelial growth factor (VEGF), a cytokine produced by tumor cells, plays an important role in angiogenesis [87]. Luteolin, a dietary flavonoid, was found to inhibit angiogenesis and the formation of vasculogenic mimicry tube in MGC-803 and Hs-746T gastric cancer cells via the suppression of notch receptor 1 (Notch1)/VEGF signaling [88]. Additionally, zerumbone, a bioactive component of ginger, showed anti-angiogenesis activity in AGS cells by decreasing the expression of VEGF via the inhibition of nuclear factor kappa light chain-enhancer of activated B cells (NF-κB) [89]. Moreover, in SGC-7901 and AGS human gastric cancer cell lines, nitidine chloride, generated from *Zanthoxylum nitidum* (Roxb) DC, was found to inhibit signal transducer and activator of transcription 3 (STAT3) signaling, which was associated with tumor angiogenesis. In a xenograft mouse model induced by SGC-7901 cells, the treatment of nitidine chloride reduced the volume of tumors via the inhibition of angiogenesis with decreased levels of STAT3 and VEGF [23].

### 3.5. Suppression of Cell Metastasis

Invasion and metastasis play a crucial role in the progression of gastric cancer [90]. Several studies found that phytochemicals could inhibit the invasion and metastasis of gastric cancer cells. It was reported that erinacine A present in *Hericium erinaceus* mycelium could inhibit the viability and invasiveness of MKN-28 and TSGH 9201 human gastric cancer cells [91]. In addition, luteolin was effective in suppressing invasion and migration by inhibiting Notch1 signaling and reversing epithelial-mesenchymal transition (EMT) in Hs-746T and MKN-28 gastric cancer cells [92]. Additionally, in SGC7901 cells, tangeretin, a polymethoxylated flavonoid of citrus fruits, inhibited radiation-mediated EMT, migration, and invasion by reducing the expression of Notch-1, two serrate-like ligands (Jagged1/2), two transcription factors (Hey-1 and Hes-1), and increasing the level of miR-410, a tumor-suppressive microRNA [93]. Moreover, gallic acid could suppress the metastasis of AGS cells through decreasing the level of matrix metalloproteinase (MMP)-2, MMP-9, and the activity of NF-κB, and downregulating PI3K/Akt pathway [90]. Gallic acid decreased the expression of RAS, but increased the expression of RhoB. Furthermore, diallyl disulfide inhibited gastric adenocarcinoma cell motility and invasiveness by increasing the tightness of tight junctions and decreasing the levels of MMP-2 and MMP-9 [94].

### 3.6. Inhibition of Helicobacter Pylori

Accumulating studies have suggested that *Helicobacter pylori* infection can cause various gastric diseases, such as chronic gastritis, peptic ulcers, and atrophic gastritis. The *Helicobacter pylori* infection is highly related to the pathogenesis of gastric cancer, particularly the intestinal type [95,96,97]. It was reported that infection with cytotoxin-associated gene antigen cagA^+^ strains of *Helicobacter pylori* might lead to severe gastric inflammation and gastric cancer [98,99]. Moreover, the growth of *Helicobacter pylori* cag*A*^+^ strains could be suppressed by curcumin and gingerols in vitro [26,100]. In NCI-N87 gastric carcinoma cells, the expression of CD74 in *Helicobacter pylori*, an adhesion molecule to urease, decreased by bergamottin, a component of citrus fruit, leading to the inhibition of *Helicobacter pylori* adhesion [101]. In addition, the treatment of apigenin, a flavonoid rich in celery, could inhibit *Helicobacter pylori* colonization, and reduce the incidence rate of gastric cancer in *Helicobacter pylori*-infected Mongolian gerbils [102]. Additionally, an in vivo study revealed that curcumin was effective in eliminating *Helicobacter pylori* from infected mice and alleviating *Helicobacter pylori*-induced gastric damage [103].

### 3.7. Modulation of Gut Microbiota

In recent years, the relationship between gut microbiota and multiple diseases has attracted much attention. The role of gut microbiota on gastric cancer has also been investigated [104,105]. A study revealed that microbiota might be related to gastric cancer, since specific pathogen-free mice were easier to develop atrophic gastritis and gastric cancer than germ-free mice [106]. It was reported that phytochemicals could prevent and manage some cancers via the modulation of gut microbiota, such as colorectal cancer, liver cancer and breast cancer [66]. However, there have been few reports about the anti-gastric cancer of phytochemicals by modulating gut microbiota, which may warrant further elucidation.

### 3.8. Adjuvant Therapy

Numerous studies have indicated that phytochemicals can enhance the sensitivity of gastric cancer to therapy, and exert a synergistic anticancer effect. A study pointed out that gartanin, a bioactive compound isolated from mangosteen, enhanced the sensitization of AGS human gastric adenocarcinoma cells to tumor necrosis factor-related apoptosis-inducing ligand (TRAIL) by increasing death receptor 5 [107]. In addition, curcumin was found to enhance the anticancer efficacy of etoposide and doxorubicin, two chemotherapeutic drugs, in SGC-7901 human gastric cancer cells by inhibiting the activation of NF-κB, and the expression of its related anti-apoptotic gene like Bcl-2 and Bcl-xL [10]. Additionally, the anticancer effects of fluorouracil and cisplatin were potentiated by genistein, an isoflavone present in soy products, which could decrease chemoresistance of MGC-803 cells through reducing the expression of adenosine triphosphate (ATP) binding cassette subfamily G member 2 (ABCG2) and the activity ERK1/2 [108]. Moreover, the combination of paclitaxel and 3,3’-diindolylmethane, a compound of cruciferous vegetables, enhanced the therapeutic efficacy via the inhibition of SNU638 cell proliferation and the induction of apoptosis, which was associated with the downregulation of the Akt/Forkhead box M1 (FOXM1) signaling [109]. Furthermore, combined treatment of diallyl trisulfide and docetaxel showed a synergistic effect against gastric cancer through inducing G_2_/M cell cycle arrest and apoptosis with increased level of metallothionein 2A (MT2A) and inhibition of NF-κB signaling in BGC823 cells [110]. In another study, diallyl trisulfide enhanced the potency of cisplatin against gastric cancer through the activation of p38 and JNK MAPK signaling pathway, and downregulation of the nuclear factor erythroid 2-related factor 2 (Nrf2)/Akt pathway in vitro and in vivo [111]. Additionally, 6-gingerol increased the cisplatin sensitivity of HGC-27 cells via the suppression of cell proliferation, migration, and invasion by inactivating PI3K/Akt signaling pathway [112].

Collectively, several phytochemicals exhibit anticancer effects against gastric cancer, such as curcumin, diallyl trisulfide, 3,3’-diindolylmethane and 6-shogaol (Table 2). The mechanisms of action are mainly inhibiting cell proliferation, inducing apoptosis and autophagy, suppressing angiogenesis and metastasis, reducing the *Helicobacter pylori* infection, and modulating the gut microbiota (Figure 2). Additionally, the combined treatment of phytochemicals and anticancer drugs exhibits synergistic effects against gastric cancer.

## 4. Clinical Trials

The efficacy of natural products against gastric cancer was also supported in clinical studies. A study reported that daily treatment of 900 mg of *Rhus verniciflua* Stokes extract decreased the polypoid mass and the flat elevated lesion in an old female patient with gastric adenocarcinoma [123]. Additionally, a randomized intervention trial including 3365 residents revealed that garlic (extract and oil) supplementation could also reduce the mortality of gastric cancer [124]. In a multi-institutional randomized prospective study, combined with clinical medicine tegafur and cisplatin, lentinan could prolong median survival and improve the quality of life in patients with gastric cancer [125]. In addition, a clinical study including 349 subjects with stage II/III gastric cancer revealed that adjuvant treatment of protein-bound polysaccharide K from the mushroom *Coriolus versicolor* could prolong the survival of major histocompatibility complex (MHC) class I-negative patients [126]. Generally, several natural products exhibited significant synergistic effects with anticancer drugs against gastric cancer. In the future, the anti-gastric cancer effects of more phytochemicals should be confirmed by clinical trials.

## 5. Bioavailability

Several phytochemicals displayed low bioavailability, such as 3,3’-diindolylmethane and curcumin [127,128,129,130]. Some technologies have been applied to increase the bioavailability of phytochemicals, which should improve the anti-gastric cancer action [131,132]. A study showed that 3,3’-diindolylmethane was microencapsulated in starch with d-α-tocopheryl acid succinate, phosphatidylcholine, and silica, which could enhance its bioavailability [127]. In addition, the pterostilbene was encapsulated in nanoemulsions containing carrier oil, which could increase its bioavailability [133]. Moreover, curcumin and genistein showed good solubility and stability after encapsulating within nanostructured lipid carriers [131]. Furthermore, the micellarization could enhance the bioaccessibility of isoflavonoid aglycones [134]. Generally, the bioavailability of phytochemicals can be increased by several methods, such as encapsulation in the nanostructured lipid carriers and micellarization.

## 6. Safety

Different from anticancer drugs, phytochemicals commonly have less toxicity, making them safer in the prevention and management of gastric cancer [83,135]. It has been reported that lentinan had low or zero toxicity, even at high doses [135]. In addition, the treatment of hispolon, which was isolated from a traditional medicinal mushroom, showed no adverse effects on human normal gastric cells [74]. In another in vivo study, no observable toxicity was found in rats with long-term exposure to 3,3’-diindolylmethane [136]. Furthermore, the extract of *Hericium erinaceus* exhibited anticancer activity against the xenograft model of NCI-87 gastric cancer cells without toxicity to the host [137]. However, some spices were found to have adverse effects. A study reported that piperine had reproductive toxicity in mice [133]. Additionally, turmeric and curcumin exhibited hepatotoxicity in mice [138].

Collectively, most experimental studies have suggested a lack or low level of toxicity of most phytochemicals. However, the toxicity and other adverse effects of some phytochemicals, such as allergic reactions, liver or kidney toxicity, have not been tested in humans. Therefore, it is necessary to determine the effective and safe doses of phytochemicals to prevent toxicity in human.

## 7. Conclusions

The effects of phytochemicals against gastric cancer have been extensively investigated. Numerous epidemiological studies have suggested that the consumption of natural dietary products such as fruits, vegetables, spices, isoflavone and quercetin is inversely related to the risk of gastric cancer. However, inconsistent results have also been reported in some cohort studies. Moreover, both in vitro and in vivo studies have revealed that some phytochemicals showed anti-gastric cancer activity by inhibiting cell proliferation, inducing apoptosis and autophagy, suppressing angiogenesis and metastasis, reducing *Helicobacter pylori* infection, and modulating the gut microbiota. In addition, phytochemicals enhanced the sensitivity to chemotherapy and had synergistic effects with anticancer drugs against gastric cancer. The clinical trials further verified the anticancer efficacy of several phytochemicals. However, the protective effects of natural products against gastric cancer by regulating gut microbiota have not yet been fully explored and understood. The effects of more natural products against gastric cancer should be evaluated, the phytochemicals should be isolated and identified, and the mechanisms of action should be elucidated. Furthermore, attention should be paid to the safety and bioavailability of phytochemicals. Overall, consumption of phytochemicals is a promising strategy for the prevention and management of gastric cancer, and the public is recommended to consume natural dietary products rich in diverse phytochemicals for the prevention of gastric cancer. These natural products could also be developed into functional foods and pharmaceuticals to prevent and treat gastric cancer.

## Figures and Tables

**Table 1:** The effects of natural dietary products against gastric cancer from epidemiological studies.

Natural Products	Phytochemicals	Subjects	Study Type	Consumed Levels	Effects	Ref.
**Fruits**						
Citrus fruits	NA	217 Gastric cancer cases (mean age: 65.4; 151 men) and controls (mean age: 64.3; 265 men) in Iran	Case-control	≥3 times/week vs. never or infrequently intake of citrus fruits	Reducing gastric cancer risk (OR, 0.31; 95% CI, 0.17–0.59)	[30]
Citrus fruits	NA	120,852 Subjects in Netherlands (58,279 men and 62,573 women), 156 gastric cardia adenocarcinoma cases and 460 gastric noncardia adenocarcinoma cases; aged 55–69 years	Cohort study	The highest (median = 156 g/d) vs. the lowest quintile (median = 0 g/d) of citrus fruits	Reducing the risk of gastric noncardia cancer (RR, 0.38; 95% CI, 0.21–0.69)	[34]
Total fruits (except watermelon)	NA	559,247 Chinese men in the cohort and 132 distal gastric cancer cases; aged 40–74 years	Cohort study	>104.2 vs. ≤20.1 g/d all fruits (except watermelon)	Reducing distal gastric cancer risk (HR, 0.50; 95% CI, 0.29–0.84)	[47]
Total fruits (except watermelon)	NA	73,064 Chinese women in the cohort and 206 distal gastric cancer cases; aged 40–70 years	Cohort study	>208.0 vs. ≤61.5 g/d all fruits (except watermelon)	No association (HR, 1.02; 95% CI, 0.68–1.54)	
Total fruit	NA	191,232 Japanese subjects, (87,771 men and 103,461 women) and 2995 gastric cancer cases (2104 men and 891 women)	Pooled analysis	The highest quintile vs. the lowest quintile of total fruit	No association (HR, 0.9; 95% CI, 0.67–1.22)	[44]
**Vegetables**						
Brassica vegetables	NA	120,852 Subjects in Netherlands (58,279 men and 62,573 women), 156 gastric cardia adenocarcinoma cases and 460 gastric noncardia adenocarcinoma cases; aged 55–69 years	Cohort study	The highest (median = 59 g/d) vs. the lowest quintile (median = 11 g/d) of Brassica vegetables	Reducing the risk of gastric noncardia cancer (RR, 0.51; 95% CI, 0.28–0.92)	[34]
Total vegetables	NA	559,247 Chinese men in the cohort and 132 distal gastric cancer; aged 40–74 years	Cohort study	>429.3 vs. ≤212.9 g/d total vegetables	No association (HR, 1.00; 95% CI, 0.59–1.68)	[47]
Total vegetables	NA	73,064 Chinese women in the cohort and 206 distal gastric cancer cases; aged 40–70 years	Cohort study	>373.7 vs. ≤179.5 g/d total vegetables	No association (HR, 0.89; 95% CI, 0.60–1.31)	
Total vegetables	NA	191,232 Japanese subjects, (87,771 men and 103,461 women) and 2995 gastric cancer cases (2104 men and 891 women)	Pooled analysis	The highest quintile vs. the lowest quintile of total vegetable	Reducing distal gastric cancer risk in men (multivariate HR, 0.78; 95% CI, 0.63–0.97)	[44]
**Fruits and vegetables**						
Fruits and vegetables	β-carotene	511 Japanese gastric cancer cases (342 men) and 511 controls (342 men); aged 40–69 years	Nested case-control	≥27.0 vs. ≤8.0 ug/dL β-carotene	Reducing gastric cancer risk (OR, 0.46; 95% CI, 0.28–0.75)	[39]
Vegetables, citrus fruits, and whole grains	NA	970,045 American subjects (533,391 women and 436,654 men) and 439 women and 910 men died from gastric cancer	Cohort study	The highest vs. the lowest tertile of plant foods	Reducing gastric cancer risk in men (RR, 0.79; 95% CI, 0.67–0.93)	[35]
Fruits, vegetables and beverages	Quercetin	505 Swedish gastric cancer cases (336 men) and 1116 controls (746 men); aged 40–79 years	Case-control	≥11.9 vs. <4 mg /day quercetin	Reducing noncardia gastric adenocarcinoma risk (OR, 0.57; 95% CI, 0.40–0.83)	[19]
**Spices**						
Allium vegetables	NA	543,220 Total subjects	Meta-analysis	The highest vs. the lowest consumption category of allium vegetables	Reducing gastric cancer risk (OR, 0.54; 95% CI, 0.43–0.65)	[36]
Garlic	NA	217 Gastric cancer cases (mean age: 65.4; 151 men) and controls (mean age: 64.3; 265 men) in Iran	Case-control	≥3 times/week vs. never or infrequently intake of garlic	Reducing gastric cancer risk (OR, 0.35; 95% CI, 0.13–0.95)	[30]
Onion	NA			≥ once per day vs. ≤2 times/week onion	Reducing gastric cancer risk (OR, 0.34; 95% CI, 0.19–0.62)	
**Soy and soy products**						
Soy	Isoflavone	84,881 Japanese subjects (39,569 men and 45,312 women), 1249 gastric cancer cases; aged 45–74 years	Cohort study	The highest vs. the lowest quartile of isoflavone	No association (HR, 1.00; 95% CI, 0.81-1.24 for men and HR, 1.07; 0.77–1.50 for women)	[49]
Soy	Isoflavone	30,792 Japanese subjects (14,219 men and 16,573 women), 678 gastric cancer cases (441 men and 237 women); aged ≥ 35 years	Cohort study	>53 vs. ≤28 mg/d isoflavone	Reducing gastric cancer risk in women (HR, 0.60; 95% CI, 0.37–0.98)	[18]
				>122 vs. ≤62 g/d soy food	Reducing gastric cancer risk in men(HR, 0.71; 95% CI, 0.53–0.96) and women (HR, 0.58; 95% CI, 0.36–0.94)	
Tofu	NA	128,687 Chinese subjects (70,446 women and 58,241 men), 493 distal gastric cancer cases; aged 40–74 years	Cohort study	>8.4 vs. <3.1 g/d tofu	Reducing distal gastric cancer risk in men (HR, 0.64; 95% CI, 0.42–0.99)	[38]
Dry bean	NA			>0.9 vs. 0.0 g/d dry bean	Reducing gastric cancer risk in postmenopausal women (HR, 0.63; 95% CI, 0.43–0.91)	
Total soy product	NA	30,304 Japanese subjects (13,880 men and 16,424 women) and 121 gastric cancer deaths; aged ≥ 35 years	Cohort study	The highest (median = 49.7 g/d) vs. the lowest tertile (median = 140 g/d) of total soy product	Reducing the risk of gastric cancer death (HR, 0.5; 95% CI, 0.26–0.93)	[28]
**Cereals**						
**Other**						
	Flavonoids	469,008 American subjects (275,982 men and 193,026 women), 1297 gastric cancer cases; aged 50–71 years	Cohort study	438.0–4211.2 vs. 0–84.1 mg/d total flavonoids	No association (HR, 1.02; 95% CI, 0.78–1.34) for gastric cardia cancer; (HR, 1.11; 95% CI, 0.86–1.44) for gastric noncardia cancer	[50]
	Flavonoids	334 Korean gastric cancer cases (208 men) and 334 controls (208 men); aged 35–75 years	Case-control study	The highest tertile (median = 152.3 mg/d) vs. the lowest tertile (median = 52.5 mg/d) of flavonoids	Reducing gastric cancer risk (OR, 0.49; 95% CI, 0.31–0.76)	[40]
	Anthocyanidins	248 American gastric cardia cancer cases and 662 controls; aged 30–79 years	Case-control study	≥18.48 vs. ≤7.21 mg/d anthocyanidins	Reducing the risk of mortality for gastric cardia cancer (HR, 0.63; 95% CI, 0.42–0.95)	[42]

NA: not available.

**Table 2:** The effects of phytochemicals against gastric cancer from experimental studies.

Natural Products	Phytochemicals	Study Type	Models	Mechanisms	Molecular Targets	Ref.
**Fruits**						
*Citrus reticulata* Blanco extract	NA	In vitro	SNU-668 cells	Induced apoptosis	↓ Bcl-2 ↑ Bax and caspase-3	[71]
*Cirsium chanroenicum*	Pectolinarigenin	In vitro	AGS and MKN-28 cells	Induced autophagy and apoptosis Inhibited cell growth and proliferation	↓ p-4EBP1, p-p70S6K, and p-eIF4E, ↑ LC3-II conversion	[82]
Citrus fruits	Poncirin	In vitro	AGS cells	Induced apoptosis Inhibited cell proliferation	↑ FasL, caspase-8, caspase-3 and PARP cleavage	[70]
Black currant	Phenolic compounds	In vitro	SGC-7901 cells		NA	[113]
Blueberries	Pterostilbene	In vitro	AGS cells		↓ p-Rb, cyclin A, cyclin E, Cdk2, Cdk4, and Cdk6, ↑ caspase-2, -3, -8, and -9, PARP cleavage, p53, p2l, p27, and p16 proteins	[114]
Citrus fruits	Tangeretin	In vitro	SGC7901 cells	Inhibited radiation-mediated EMT, migration and invasion	↓ Notch-1, Jagged1/2, Hey-1 and Hes-1, ↑ miR-410	[93]
Mangosteen	α-Mangostin	In vitro	BGC-823 and SGC-7901 cells	Induced apoptosis Inhibited the cell viability	↓ STAT3, Bcl-xL and Mcl-1, ↑ cytochrome c	[72]
Mangosteen	Gartanin and TRAIL	In vitro	AGS cells	Enhanced the sensitization of AGS cells to TRAIL	↑ death receptor 5	[107]
Strawberry	NA	In vitro	SNU-638 cells	Inhibited cell growth	NA	[115]
*Citrus reticulate* cv. Suavissima	Poncirin	In vitro	SGC-7901 cells			[54]
**Vegetables**						
Cruciferous vegetables	3,3’-Diindolylmethane	In vitro	BGC-823 and SGC-7901 cells	Inhibited cell proliferation Induced autophagy	↓ MicroRNA-30e, ↑ ATG5 and LC3	[83]
		In vivo	Female nude mice	Inhibited the growth of gastric tumor	↑ LC3	
Cruciferous vegetables	Paclitaxel and 3,3’-diindolylmethane	In vitro	SNU638 cell	Induced apoptosis Inhibited proliferation	↑ PARP, caspase-9, ↓ CDK4, p53, cyclin D1 and p-Akt	[109]
**Spices**						
Fruit of long pepper	Piperlongumine	In vitro	SGC-7901, BGC-823 and KATO III cells	Induced apoptosis	↓ TrxR1, ↑ ROS	[21]
		In vivo	Female BALB/cA athymic mice	Reduced tumor cell burden	↓ TrxR1	
Allitridi	NA	In vitro	BGC823 cells	Induced apoptosis Inhibited cell proliferation	↓ Bcl-2, ↑ caspase-3	[52]
*Allium ursinum* L	NA	In vitro	AGS cells		↓ cyclin B	[56]
Garlic	Diallyl trisulfide	In vitro	AGS cells		↑ ROS, phosphorylation of AMPK and histone H3	[60]
Ginger	6-Shogaol	In vitro	HGC, AGS and KATO III cells	Inhibited cell viability Induced mitotic arrest Damaged microtubules	NA	[64]
		In vivo	Athymic nude mice	Suppressed tumor growth	NA	
Ginger	Zerumbone	In vitro	AGS cells	Anti-angiogenesis	↓ VEGF and NF-κB	[89]
Ginger	6-Gingerol and cisplatin	In vitro	HGC-27 cells	Inhibited cell proliferation, migration and invasion	↑ P21 and P27, ↓ cyclin D1, cyclin A2, MMP-9, p-PI3K, Akt, and p-Akt	[112]
*Curcuma zedoaria* rhizomes	Curcuzedoalide	In vitro	AGS cells	Induced apoptosis Inhibited cell viability	↑ cleavage of caspase-8, caspase-9, caspase-3 and PARP	[116]
*Curcuma mangga* rhizomes	Labdane diterpenes	In vitro	AGS cells	Inhibited cell proliferation	NA	[53]
Turmeric	Curcumin, etoposide and doxorubicin	In vitro	SGC-7901 cells	Enhanced the anticancer efficacy of etoposide and doxorubicin	↓ NF-κB, Bcl-2 and Bcl-xL	[10]
Garlic	Diallyl trisulfide and docetaxel	In vitro	BGC823 cells	Induced apoptosis Induced G_2_/M cell cycle arrest	↑ MT2A, IκB-α, cyclin B1, activated caspase-3, and Bax, ↓ p-IκB-α, p-P65, cyclin D1, and XIAP	[110]
		In vivo	Female BALB/c athymic mice	Inhibited tumor growth	↑ MT2A, IjB-a, CCNB1, and a-CASP3, ↓ CCND1	
Garlic	Diallyl disulfide	In vitro	MGC803 cells	Inhibited cell growth Induced cell differentiation	↓ CDC25C, cyclin B1, p-ERK1/2, ↑ p-Chkl	[57,59]
Garlic	Diallyl disulfide	In vitro	AGS cells	Inhibited tumor cell motility and invasion	↓ MMP-2, MMP-9, claudin proteins (claudin-2, -3, and -4), ↑ TIMP-1, TIMP-2	[94]
Garlic derivatives	S-allylmercaptocysteine	In vivo	Female BALB/c nude mice	Inhibited the growth of gastric tumor	NA	[63]
*Zanthoxylum nitidum (Roxb) DC*	Nitidine chloride	In vitro	SGC-7901 and AGS cells	Induced apoptosis Inhibited cell viability and angiogenesis	↓ p-STAT3, cyclin D1, Bcl-2, Bcl-xL, and VEGF	[23]
		In vivo	Male BALB/cA nude mice	Reduced the volume of tumors	↓ STAT3 and VEGF	
**Mushroom**						
Liang Jin mushroom	3’-azido-3’-deoxythymidine (AZT) and RNA-protein complex (FA-2-b-β)	In vitro	MKN-45 cells	Induced apoptosis Inhibited cell proliferation	↓ tumor cell telomerase and Bcl-2, ↑caspase-3	[117]
*Agaricus blazei* Murrill	Blazein	In vitro	KATO III cells	Induced apoptosis Suppressed cell growth	NA	[118]
*Phellinus linteus*	Polyphenol compound hispolon	In vitro	SGC-7901, MGC-803, and MKN-45 cells	Induced apoptosis	↓ Bcl-2, ↑ ROS, cytochrome c, caspase-3 and caspase-9	[74]
*Hericium erinaceus* mycelium	Erinacine A	In vitro	TSGH9201 and MKN-28 human gastric cancer cells	Induced apoptosis Inhibited the viability and invasiveness	↓ Bcl-2 and Bcl-XL, ↑ ROS, MTUS2, TRAIL, caspase 8, caspase 9, caspase 3, cytochrome c and phosphorylation of FAK/Akt/p70S6K and PAK1	[91]
*Lentinula edodes* C91-3	Latcripin 1 protein	In vitro	SGC-7901 and BGC-823 cells	Induced autophagy and apoptosis Inhibit cell growth and proliferation	↓ Bcl-2, MMP-2 and MMP-9, ↑ Bax, caspase-3, ATG7, ATG5, ATG12, ATG14 and Beclin1	[61]
*Ganoderma lucidum*	NA	In vitro	AGS cells		↑ LC3-II	[119]
	Recombinant Lz-8 protein	In vitro	SGC-7901 cells	Induced autophagic cell death Inhibited cell growth	↑ CHOP, ATF4 and GRP78	[120]
*Fomes Fomentarius*	Polysaccharide	In vitro	SGC-7901 and MKN-45 cells	Inhibited cell proliferation	NA	[20]
Maitake (*Grifola frondosa*)	NA	In vitro	TMK-1, MKN-28, MKN-45 and MKN-74 cells		NA	[121]
**Soy**						
Black soybean	NA	In vitro	AGS cells	Induced apoptosis Inhibited cell proliferation	↓ Bcl-2, ↑ Bax, caspase-3, PARP cleavage	[75]
Soy products	Genistein, fluorouracil and ciplatin	In vitro	MGC-803 cells	Decreased chemoresistance	↓ ABCG2, ERK1/2	[108]
**Traditional medicine**						
*Gardenia jasminoides* Ellis	Carotenoids	In vitro	MKN-28 cells	Inhibited cell proliferation	NA	[122]
*Perilla frutescens*	Perillaldehyde	In vitro	MFCs and GC9811-P cells	Induced autophagy	↑ p-AMPK	[84]
		In vivo	Female BAL B/c nude mice	Inhibited the growth of gastric tumor Induced autophagy	↑ beclin-1, LC3-II, cathepsin, caspase-3 and p53	
*Vitex agnus-castus* fruit	NA	In vitro	KATO-III Cells	Induced apoptosis	↓ Bcl-2, Bcl-XL, Bid, Mn-superoxide dismutase and catalase, GSH, ↑ Bad, cytochrome c, caspase-3 caspases-8, caspases-9, hemeoxygenase-1 and thioredoxin reductase	[73]
Bamboo shavings	Polysaccharides	In vivo	Syngeneic murine gastric cancer model	Inhibited tumor growth Prolonged the survival	↑ cleaved caspase 3, Bax and Bik	[79]
**Other**						
	Protocatechuic acid	In vitro	AGS cells	Induced apoptosis Inhibited cell proliferation	↓ cyclin B, ↑ JNK and p38 MAPK	[69]
	Kaempferol	In vitro	AGS, NCI-N87, SNU-638 and MKN-74 cells	Induced autophagic cell death Decreased cell viability	↓ p62, ↑ LC3B, Beclin-1, ATG5, p-IRE1 and p-JNK	[22]
	Myricetin	In vitro	HGC-27 and SGC7901 cells	Inhibited cell proliferation	↑ Mad1	[62]
	Apigenin	In vitro	SGC-7901 cells	Inhibited cell growth	NA	[55]
	Luteolin	In vitro	Hs-746T and MKN-28 cells	Induced cell apoptosis Inhibited cell proliferation, invasion, and migration	↓ Notch1	[92]
		In vivo	Male BALB/c nude mice	Reduced gastric tumor volume and tumor weight	↓ β-catenin, Notch1 and Ki-67	
	Gallic acid	In vitro	AGS cells	Inhibited cell metastasis	↓ MMP-2, MMP-9, NF-κB, Ras, Cdc42, Rac1, RhoA, RhoB and PI3K	[90]
	Luteolin	In vitro	MGC-803 and Hs-746T cells	Anti-angiogenesis Inhibited the formation of vasculogenic mimicry tube	↓ VEGF and Notch1	[88]
	Quercetin and SN-38 (a metabolite of irinotecan)	In vivo	Female BALB/c nude mice	Reduced the volume of tumors Anti-angiogenesis and anti-metastasis	↓ cyclooxygenase-2, Twist1, ITGβ6, VEGF-R2 and VEGF-A	[24]
		In vitro	AGS cells	Induced apoptosis	↓ β-catenin	

NA: not available.

## Abbreviations

AMPK	AMP-activated protein kinase
ATF4	activating transcription factor 4
ATG5	autophagy related 5; ABCG2
ABCG2	ATP binding cassette subfamily G member 2
Bad	Bcl-2 associated agonist of cell death
Bax	Bcl-2-associated X protein
Bcl-2	B-cell lymphoma 2
Bcl-xL	B-cell lymphoma-extralarge
Bid	BH3 interacting domain death agonist
Bik	Bcl-2 interacting killer
Cdc42	cell division cycle 42
CDC25C	cell division cycle 25C
CDK4	cyclin dependent kinase 4
CHOP	-CCAAT-enhancer-binding protein homologous protein
EMT	epithelial-mesenchymal transition
ERK1/2	extracellular signal-regulated kinase
FasL	Fas Ligand
GRP78	glucose regulated protein 78
GSH	glutathione
Hes-1	hes family bHLH transcription factor 1
Hey-1	hes related family bHLH transcription factor with YRPW motif 1
IκB-α	inhibitor of NF-κB
ITGβ6	integrin subunit beta 6
Jagged1/2	2 serrate-like ligands
JNK	c-Jun N-terminal kinase
ki-67	a cell proliferation marker
LC3B	microtubule associated protein 1 light chain 3 beta
Mad1	Mitotic arrest-deficient 1
MAPK	mitogen-activating protein kinase
Mcl-1	apoptosis regulator blongs to Bcl-2 family member
miR-410	a tumor-suppressive microRNA
MMP-2	matrix metallopeptidase 2
MT2A	metallothionein 2A
MTUS2	microtubule-associated tumor suppressor candidate 2
NF-κB	nuclear factor kappa light chain-enhancer of activated B cells
Notch1	notch receptor 1
PARP	poly (ADP-ribose) polymerase
p-ERK1/2	phosphorylation of extracellular signal-regulated kinase
p-Chkl	phosphorylation of checkpoint kinase-1
p-IRE1	phosphorylates inositol-requiring-1
p-JNK	phosphorylates c-Jun N-terminal protein kinase
p-4EBP1	phosphorylated 4E binding protein 1
p-p70S6K	phosphorylated ribosomal protein S6 kinase
p-eIF4E	phosphorylated eukaryotic translation initiation factor 4E
PI3K	phoshatidylinositol-3-kinase
p-IκB-α	phosphorylation of p-IκB-α
Rac1	Rac family small GTPase 1
RhoA	ras homolog family member A
RhoB	ras homolog family member B
ROS	reactive oxygen species
STAT3	signal transducer and activator of transcription 3
TIMP	tissue inhibitor of metalloproteinase
TrxR1	thioredoxin reductase 1
TRAIL	tumour necrosis factor (TNF)-related apoptosis-inducing ligand
Twist1	twist family bHLH transcription factor 1
VEGF	vascular endothelial growth factor
VEGF-R2	vascular endothelial growth factor receptor 2
XIAP	X-linked inhibitor of apoptosis protein
